# Self-control, implicit alcohol associations, and the (lack of) prediction of consumption in an alcohol taste test with college student heavy episodic drinkers

**DOI:** 10.1371/journal.pone.0209940

**Published:** 2019-01-09

**Authors:** Kristen P. Lindgren, Scott A. Baldwin, Jason J. Ramirez, Cecilia C. Olin, Kirsten P. Peterson, Reinout W. Wiers, Bethany A. Teachman, Jeanette Norris, Debra Kaysen, Clayton Neighbors

**Affiliations:** 1 Department of Psychiatry & Behavioral Sciences, University of Washington, Seattle, Washington, United States of America; 2 Department of Psychology, Brigham Young University, Provo, Utah, United States of America; 3 Department of Psychology, University of Memphis, Memphis, Tennessee, United States of America; 4 Department of Psychology, University of Amsterdam, Amsterdam, The Netherlands; 5 Department of Psychology, University of Virginia, Charlottesville, Virginia, United States of America; 6 Alcohol & Drug Abuse Institute, University of Washington, Seattle, Washington, United States of America; 7 Department of Psychology, University of Houston, Houston, Texas, United States of America; Univerity of Salzburg, AUSTRIA

## Abstract

The high levels of problematic drinking in college students make clear the need for improvement in the prediction of problematic drinking. We conducted a laboratory-based experiment that investigated whether implicit measures of alcohol-related associations, self-control, and their interaction predicted drinking. Although a few studies have evaluated self-control as a moderator of the relationship between implicit measures of alcohol-related associations and drinking, this study extended that work by using a previously-validated manipulation that included a more (vs. less) cognitively demanding task and incentive to restrain drinking and by evaluating multiple validated measures of alcohol-related associations. Experimental condition was expected to moderate the relationship between implicit measures of alcohol-related associations and drinking, with a more positive relationship between alcohol-related associations and drinking among participants who completed the more (vs. less) cognitive demanding task. Secondary aims were to evaluate how individual differences in control factors (implicit theories about willpower and working memory capacity) might further moderate those relationships. One hundred and five U.S. undergraduate heavy episodic drinkers completed baseline measures of: drinking patterns, three Implicit Association Tests (evaluating drinking identity, alcohol excite, alcohol approach associations) and their explicit measure counterparts, implicit theories about willpower, and working memory capacity. Participants were randomized to complete a task that was more (vs. less) cognitively demanding and were given an incentive to restrain their drinking. They then completed an alcohol taste test. Results were not consistent with expectations. Despite using a previously validated manipulation, there was no evidence that one condition was more demanding than the other, and none of the predicted interactions reached statistical significance. The findings raise questions about the relation between self-control, implicit measures of alcohol-related associations, and drinking, as well as the conditions under which implicit measures of alcohol-related associations predict alcohol consumption in the laboratory.

## Introduction

The quantity and frequency of alcohol consumption and associated consequences remain extremely high in U.S. college students. For example, 33% of U.S. college students report at least one instance of consuming 5 or more drinks on at least one occasion in the previous two weeks, and 10% report at least one instance of consuming 10 or more drinks on one occasion in the previous two weeks [1]. Further, college student drinking is associated with frequent, substantial negative consequences, including sexual and physical assault, blackouts, poor educational performance, relationship problems, injuries, and death [2,3]. Despite progress in identifying risk factors for problematic drinking in college students and developing efficacious interventions [3–5], the persistently high levels of problematic drinking in college students make clear the need for further improvement in the prediction of problematic drinking and, ultimately, the identification of additional prevention and intervention targets. The current study sought to do so by way of a laboratory-based experiment that investigated implicit measures of alcohol-related associations in interaction with self-control as potential factors that could predict actual college student drinking in the moment.

### Implicit measures of alcohol-related associations

The use of implicit measures of alcohol-related associations has roots in dual process models of substance use [6–8]. These measures, while not process-pure, are thought to assess memory representations that influence behavior. Although dual process models are undergoing revision and reformulation [9,10], studies have shown that implicit measures of alcohol-related associations predict college students’ drinking over time and do so over and above their self-report measure counterparts [10–13]. However, the majority of studies that include implicit measures have focused on predicting self-reported retrospective drinking (e.g., self-reported drinking over the last two weeks or three months). Predicting actual drinking behavior during a discrete period or session—whether in the laboratory or natural environment—has been understudied. This is problematic for several reasons. First, while self-report measures of drinking are well validated, they—like any self-report measures—can be affected by self-presentation and/or memory biases [14]. Second, experimental laboratory studies can support causal inferences and are a key way to study mechanisms that are posited to underlie behavior. Third, theories that emphasize the role of implicit measures of alcohol-related associations in alcohol consumption delineate factors that are posited to lead to drinking on a *given occasion* versus predicting *aggregated drinking behavior over time* [8]. Studies assessing moderators of implicit measures of alcohol-related associations are also relatively scant, particularly in the context of predicting drinking during a discrete period or session. This gap is critical because theories emphasizing implicit processes stipulate that implicit processes should be stronger predictors of drinking under certain conditions (e.g., when self-control is taxed; [15]). Thus, the current study evaluated whether implicit measures of alcohol-related associations add to the prediction of drinking in the laboratory, particularly when individuals have engaged in a cognitively demanding task and have an incentive to exert self-control (i.e., to restrain their drinking).

Alcohol-related associations assessed by implicit measures are part of a broader class of implicit processes and refer to constructs related to alcohol that are held in memory; that are linked; and that do not require conscious reflection to influence affect, cognition, or behavior ([10], but see [16] for a dissenting view). These associations can be measured indirectly via adaptations of the Implicit Association Test [17], a computerized task. Performance on the IAT is thought to index the strength of associations between mental constructs by measuring the relative speed at which participants are able to classify stimuli into superordinate categories. Findings from multiple meta-analyses indicate that scores on IATs related to alcohol and drinking have small but significant effects in predicting drinking behaviors cross-sectionally [11,13,18]. Further, there is now growing evidence that IAT scores predict college student drinking both cross-sectionally and longitudinally [19–21], above and beyond conceptually-related self-report counterpart measures [20,21] and other well-established cognitive risk factors [22]. Although we know IAT scores are associated with self-reported, aggregated drinking behavior to varying degrees [20,21], we know less about whether IAT scores predict individual instances of drinking, particularly those that do not rely on self-report (e.g., *in vivo* alcohol consumption in the laboratory).

Models of alcohol misuse make predictions about conditions under which implicit processes should better predict drinking [15], such as when drinking is more (vs. less) habitual or under certain mood states. According to Hofmann and colleagues’ model [15], implicit processes should also be more influential when cognitive resources are taxed and when individuals need to exert self-control. Thus, there should be a stronger, more positive, relation between alcohol-related associations (as measured by the IAT) and drinking under conditions where individuals have engaged in a more (vs. less) cognitively demanding task (e.g., completing a task that requires attending to more vs. fewer instructions) and when individuals have an incentive to restrain their drinking. In contrast, cognitive processes that require reflection and introspection (as measured by self-report questionnaires) should be more strongly and positively related to drinking under conditions where individuals have engaged in a less (vs. more) cognitively demanding task and have an incentive to restrain their drinking. However, examination of these moderating conditions, particularly in the context of laboratory studies has been limited. This is particularly unfortunate because we suspect it is a situation that college student drinkers routinely face (e.g., imagine a student who completes a cognitively demanding day of classes, is presented with an opportunity to drink, and simultaneously has an incentive to exert self-control and restrain her drinking because she needs to study for an upcoming exam).

Several laboratory studies have examined the effects of cognitive demands and incentives to restrain drinking on alcohol consumption. Collectively, they have shown that drinkers consume more alcohol in the lab after completing more demanding tasks relative to less demanding tasks (e.g., viewing negatively-valenced images and completing a thought listing task either with instructions to suppress negative emotions—more demanding, or with no instructions to suppress emotions—less demanding) when there is incentive to restrain drinking [23–25]. An additional study found that underage social drinkers were more likely to violate self-imposed drinking limits in their natural environment on days when they experienced higher demands (e.g., dealt with stress, tried to control their thoughts) relative to their own averages [26]. With regard to the role of alcohol-related associations, two studies demonstrated a positive, significant relation between alcohol-related associations and alcohol consumption only among individuals who completed more (vs. less) demanding tasks [24,27]. These studies also included self-report measure counterparts to implicit measures, which we henceforth refer to as explicit measures for brevity, but varied with regard to their findings. One study found that a positive, significant relation between an explicit measure of alcohol attitudes and alcohol consumption only among individuals who completed a less (vs. more) demanding task [27], and the other study found that condition did not moderate the relation between explicit measure and consumption [24].

Considering these studies’ mixed findings and methodologies collectively, two important areas for future research emerge. First, two of the previous studies included implicit measures assessing appetitive inclinations towards alcohol, whether via an IAT assessing associations between alcohol and approach [23] or via a different task (the Stimulus Response Compatibility task) assessing how quickly one moves a mannikin toward or away from images with or without alcohol [24]. The third study included an IAT assessing associations between alcohol and valence (i.e., good/bad, [27]). There are other alcohol-related associations that, based on recent studies [19,20], also predict U.S. college student drinking but have rarely been examined in interaction with more or less cognitive demands and incentives to exert self-control. We sought to address this gap by including an IAT assessing alcohol approach associations (because it is more commonly studied in this domain and, thus, serves a partial replication study) *and* two other IATs assessing alcohol-related associations that have strong support as predictors of U.S. college student drinking [21,22,26]. Specifically, we examined associations between drinking and the self (i.e., drinking identity [20,21]) and between alcohol and excitement [19,20]. We note that there is some evidence that the latter is also associated with college students’ alcohol consumption in the laboratory [27].

Second, the manipulations in previous studies also involved inducing and, depending upon condition, suppressing negative emotions [23] or controlling one’s emotions [25]. Given theory that postulates [15] and laboratory experimental studies that demonstrate [27,28] that mood also can moderate the relation between implicit processes and alcohol consumption, it is important to test whether more or less cognitively demanding tasks and incentive to exert self-control over drinking, *in the absence of a mood induction*, moderates the relationship between alcohol-related associations and drinking. Consider that college students—via engaging in many of the behaviors central to being students (e.g., attending classes, completing assignments, and studying)–are likely regularly engaging in cognitively demanding tasks that may not induce affect. It is important to understand how those kinds of experience do or do not relate to their drinking. Thus, in the current study we used an experimental manipulation that varied in its cognitive demand (i.e., it had more vs. less complex instructions) but that was not intended to induce or suppress a strong affective state [29].

### Controversy over the effects of exerting self-control

The underlying premise of several studies reviewed above is that cognitive (and/or affective) resources are limited and that engaging in cognitively (and/or affectively) demanding tasks results in the depletion of those resources. This premise links to a larger phenomenon referred to as ego depletion [29,30], which stipulates that that individuals who exert effort on one task will perform worse on a subsequent task that requires self-control. Ego depletion has become controversial. On the one hand, there is a large literature demonstrating findings consistent with the ego-depletion hypothesis, including a meta-analysis [31]. On the other hand, findings from two more recent meta-analyses [32,33] and a recent analysis of arguments from the larger debate [34] raise concerns about the magnitude of the effect size for ego depletion and whether the ego depletion effect is a “real” phenomenon. Of particular concern, a large-scale, pre-registered replication study of a standardized ego depletion protocol [35] found null results.

Because of these contrasting findings and the larger controversy, we included and evaluated two additional potential moderators. First, there are findings that individuals’ beliefs about whether self-control is limited (vs. unlimited) moderate ego depletion effects [36]. For example, Job and colleagues found that effects consistent with the ego depletion hypotheses were limited to individuals who held beliefs (referred to as “implicit theories about willpower”) that self-control has limited capacity. Findings from later studies suggest that having beliefs that self-control is limited is problematic and is associated with negative real-world outcomes, such as poor time management and lower grades [37,38]. Given these findings, we posited that the stronger relationship between alcohol-related associations and alcohol consumption hypothesized for individuals who engaged in a more (vs. less) cognitively demanding task and have an incentive to exert self-control over (i.e., restrain) their drinking might be specific to those individuals who believe that self-control has limited capacity. Thus, we proposed a 3-way interaction between alcohol-related associations, self-control (we use this term for brevity to refer to the combination of the experimental condition and the incentive to restrain drinking), and implicit theories about willpower.

Second, theory and findings suggest that individual differences in cognitive capacity (for example, working memory capacity) moderate the relationship between implicit processes and behaviors, including drinking [15,39–41]. We therefore posited that individual differences in cognitive capacity might further differentiate the extent to which self-control moderates the relation between alcohol-related associations and increased drinking. Specifically, we hypothesized that there would be a stronger, more positive relation between alcohol-related associations and alcohol consumption for individuals who engaged in a more (vs. less) cognitively demanding task combined with an incentive to restrain their drinking among participants with generally lower (vs. higher) levels of cognitive capacity. Thus, we proposed a 3-way interaction between alcohol-related associations, self-control, and a measure of working memory capacity [42].

### Study overview and hypotheses

Our primary aim was to test whether there was a stronger positive relationship between alcohol-related associations and alcohol consumption during a drinking session (that included an *ad libitum* alcohol taste test) in the laboratory in participants who completed a task that was more versus less cognitively demanding. All participants were given an incentive to restrain their drinking in the taste test; specifically, they were offered the chance to win a prize based on their performance on a task that would immediately follow. We evaluated two additional factors as moderators of the alcohol-related association by self-control relation: implicit theories about willpower and working memory capacity. For both factors, we expected a 3-way interaction, such that the aforementioned alcohol-related association x self-control interaction would be specific to individuals who believe that self-control is a limited resource or individuals with lower working memory capacity. The pattern of findings described above was expected to be the same across all three alcohol-related associations. Explicit (self-report) measure counterparts to the alcohol-related associations were also evaluated. Consistent with theory [15] and previous studies [23,25], the opposite pattern of findings was expected for explicit measures, self-control, and drinking. That is, we expected that there would be a stronger positive relationship between explicit measure counterparts and alcohol consumption in participants who completed a task that was less (vs. more) cognitively demanding amidst having an incentive to restrain their drinking.

## Materials and methods

### Participants

Participants included 105 students (55 identified their birth sex as male, 50 as female) at a large public university in the U.S. who reported consuming 4/5 or more drinks for women/men on at least one occasion in the past 30 days. All participants were required to be at least 21 years of age (*M* = 21.28, *SD* = 0.6) and in their third year of school or above. Four percent of participants identified as Hispanic or Latino. Sixty-four percent identified as white, 26% as Asian, 6% as more than one race, 3% as Native Hawaiian/Pacific Islander or American Indian/Alaskan Native, and 1% declined to answer. Four participants were excluded from analyses: one due to a computer malfunction, one due to distraction (i.e., searching the internet) during the assessment, one due to possible intoxication from a substance other than alcohol, and one due to a protocol deviation (i.e., an experimenter error in administering the protocol). Final analyses included 101 participants. Data for all participants included in analyses are publicly available at the Open Science Framework: https://osf.io/bg75v/

Sample size was determined based on findings from Ostafin and colleagues [23]. We expected the two-way interactions between the experimental conditions and alcohol associations to be in the small to medium range. Power analyses were conducted using GPower [43]. A sample size of 100 (50 per experimental condition) was expected to provide.80 power to detect the predicted two-way interactions (which were expected to account for a 7% increment in proportion of variance accounted for). We exceeded sample estimates by five participants because of two factors. First, during data collection, two participants experienced a computer malfunction and one experienced protocol deviation, and we knew their data would not be usable. Second, due to a history of no-shows for the laboratory session, we scheduled extra sessions for our final week of data collection and had a slightly higher rate of participation than expected.

### Measures

#### Alcohol-related associations

The Implicit Association Test (IAT; [17]) was used to evaluate participants’ alcohol associations. The present study included three variants of the IAT to evaluate the strength of participants’ associations with drinking and the self (drinking identity IAT; [21]), alcohol and approach (alcohol approach IAT; [44]), and alcohol and excitement (alcohol excite IAT; [21,45]).

The IAT is a computer-based reaction time (RT) task. It requires participants to classify stimuli rapidly into superordinate categories. The strength of participants’ associations between those categories is posited to be indexed by the relative speed at which participants classify stimuli into categories when the categories are paired to match vs. contradict their involuntary associations between those categories. Each IAT included two sets of contrasting target and attribute categories. Using the drinking identity IAT as an example, participants classify stimuli representing two identity categories: “me” and “not me” (i.e., target categories), as well as stimuli representing two drinking categories: “drinker” and “non-drinker” (i.e., attribute categories). The following category labels (italicized) and stimuli are the same as those used in previous studies [19–21]: drinking identity IAT *drinker*: drinker, partier, drink, drunk; *non-drinker*: non-drinker, abstainer, sober, abstain; *me*: me, my, mine, self; *not me*: they, them, theirs, other; alcohol approach IAT *alcohol*: pictures of alcohol; *water*: pictures of water; *approach*: approach, closer, advance, forward, toward; *avoid*: avoid, away, leave, withdraw, escape; and alcohol excite IAT *alcohol*: pictures of alcohol; *water*: pictures of water; *excite*: cheer, fun, high, amplify, excite; *depress*: sedate, deplete, lessen, depress, quiet. The same alcohol pictures were used in the alcohol approach and alcohol excite IATs. Participants chose a selection of four pictures (out of 12) that best represented the kinds of alcohol that they typically consumed. Stimuli were self-made and formed a standardized set that have been used in multiple, published studies [20,21]. The alcohol stimuli chosen using this method correspond well with college students’ alcohol preferences, as a function of their gender and level of drinking [46]

The IATs used the traditional seven-block structure [17]. During each block, participants completed trials in which a single stimulus appeared in the center of the screen. As quickly as possible, participants classified the stimulus into one of the categories, which were listed on the left and right sides of the screen. Participants indicated their classification using designated keys (*e* for left and *i* for right). Blocks 1, 2, and 5 were practice blocks, during which only one set of categories was listed on the screen (e.g., drinker and non-drinker *or* me and not me). All remaining blocks (3, 4, 6, & 7) were test blocks and included all categories, with one target *and* one attribute category paired on each side of the screen. During these blocks, participants classified the stimuli based on the paired target and attribute categories. Participants were given feedback about errors and had to correct an error before moving on to the next trial. There was no time limit for responses. Each pairing represented an association between the two categories, and faster responses indicate a stronger association. In the drinking identity IAT, for example, blocks 3 and 4 might pair “me” with “drinker” on the left and “not me” and “non-drinker” on the right. During blocks 6 and 7, this would switch, with “me” and “non-drinker” on the left and “not me” and “drinker” on the right. Faster responses during blocks 3 and 4 would indicate a stronger association between “me” and “drinker” as well as “not me” and “non-drinker.” To reduce the possibility of order effects, the blocks in which each target-attribute pairing was presented were counterbalanced across participants and the order in which participants completed the three IATs was randomized. To prevent participant fatigue, the IATs were interspersed among the self-report measures in the assessment battery.

The IATs were scored using the D_1_ scoring algorithm described in detail by Greenwald and colleagues (see [47] p. 210, Table 2, D_1_ column). The IAT D_1_ score (henceforth referred to simply as a D score) consists of the difference between the mean latency in blocks 3 and 4 and the mean latency in blocks 6 and 7, divided by standard deviation in blocks 3, 4, 6, and 7. The resulting D score indicates the standardized difference in average response time (i.e., latency) across each pairing and is posited to index the relative strength of each association. As such, higher scores indicated faster response times and thus a stronger association for one pairing relative to the other pairing. The drinking identity, alcohol approach, and alcohol excite IATs were scored such that higher scores indicated faster response times when pairing “drinker” and “me,” “alcohol” and “approach,” and “alcohol” and “excite,” respectively. Nosek and colleagues [48] recommend that IAT scores be excluded for individuals with errors on 30% or more trials or individuals who complete 10% or more trials in less than 300 milliseconds. Based on these criteria, four alcohol approach and three drinking identity scores were excluded. To evaluate internal consistency for each IAT [47], two D scores were calculated, one for blocks 3 and 6 and one for blocks 4 and 7, and then the D scores were correlated with one another. Internal consistency in the present study was typical for alcohol-related IATs [21]: *r* =.54 for drinking identity, *r* =.43 for alcohol approach, and *r* =.60 for alcohol excite.

#### Explicit counterparts to the alcohol-related associations

*Explicit drinking identity* was assessed using the Alcohol Self-Concept Scale (ASCS; [21]), adapted from the Smoker Self-Concept Scale [49]. The ASCS includes five items examining the role that drinking plays in one’s life and personality (e.g., “Drinking is part of who I am”). Participants rated their agreement on a 7-point scale, from *strongly disagree* (1) to *strongly agree* (7). Cronbach’s alpha was.83. Mean scores on the five items were calculated. Due to considerable positive skew and consistent with practices of Lindgren et al. [22], mean scores were dichotomized such that a score of 0 indicated absolutely no endorsement of drinking identity at all (mean score of 1) and a score of 1 indicated anything other than no endorsement (mean score > 1).

*Explicit alcohol approach* was evaluated using the inclined/indulgent subscale of the Approach and Avoidance of Alcohol Questionnaire (AAAQ; [50]). This subscale includes five items regarding participants’ inclinations to approach alcohol in the past week (e.g. “I would have liked to have a drink or two”). Participants rated their agreement with each item on a 9-point scale from *not at all* (0) to *very strongly* (8). Cronbach’s alpha was.75.

*Explicit alcohol excite* was measured using the enhancement subscale of the Drinking Motives Questionnaire (DMQ; [51]). This subscale includes five items assessing the extent to which one drinks to enhance one’s mood (e.g. “Because it gives you a pleasant feeling”). Participants responded on a 5-point scale from *never/almost never* (1) to *almost always/always* (5). Cronbach’s alpha was.84.

#### Implicit theory of willpower

The Implicit Theories about Willpower measure ([36], Study 1) evaluated the extent to which participants conceptualized willpower as a limited (e.g. “After strenuous mental activity, your energy is depleted and you must rest to get it refueled again”) or unlimited resource (e.g. “Your mental stamina fuels itself. Even after strenuous mental exertion, you can continue doing more of it”). The measure includes six items rated on 6-point Likert scales from *strongly agree* (1) to *strongly disagree* (6). Higher scores indicate conceptualization of willpower as an unlimited resource. Cronbach’s alpha was.81.

#### Working memory capacity

Working memory capacity was examined using an automated version of the Operation Span Task [42,52]. During this task, participants are presented with alternating letters and arithmetic problems (e.g. 1 + 6 ÷ 3 = 3). To move forward, participants were required to indicate whether the answer listed in the arithmetic problem was correct or incorrect using designated key presses (left arrow if correct, right arrow if incorrect). Participants were informed they would have 5 seconds to solve each math problem; otherwise the problem would be counted as incorrect. Accuracy rates were not communicated explicitly to participants. After each arithmetic problem, a letter was flashed on the screen for two seconds before a new arithmetic problem appeared. The objective was to memorize the presented letters sequentially. The OSPAN includes 12 sets of problems and letters, each of which includes 5 trials that alternate between solving an arithmetic problem and viewing a letter. At the end of each set, participants indicate the order in which the five letters were presented. Participants were shown a screen with four letter options and asked to indicate which letter appeared first in that set. Once selected, the screen showed four new letters and participants selected the second letter that appeared during the set. This continued until they had indicated all of the five letters presented in order. The first set of trials was practice to allow participants to become familiar with this procedure. The remaining sets were used in generating the OSPAN score used in the present study.

The primary OSPAN variable is the total number of correct letter sets (i.e., sets in which all five letters are correctly identified) for individuals who completed the math problems with a minimum of 80% accuracy. Scores for individuals who had less than 80% accuracy were considered invalid and were screened out [52]. Based on this criterion, nine individuals’ scores were screened out.

#### Alcohol consumption and problems

*Daily alcohol consumption* was evaluated using the Daily Drinking Questionnaire (DDQ; [53]), which examines daily alcohol consumption during a typical week in the last three months. Participants indicated the number of standard alcoholic drinks that they would typically consume on each day of the week. U.S. standard drink equivalencies were provided (e.g. 12 oz. beer, 10 oz. microbrew beer, 5 oz. wine, 1.5 oz. 80-proof hard liquor).

*Hazardous drinking* was assessed using the Alcohol Use Disorder Identification Test (AUDIT; [54]). The AUDIT evaluates risk of developing an alcohol use disorder by assessing individuals’ drinking behavior and related consequences. The AUDIT includes 10 items about alcohol consumption, alcohol dependence, and negative consequences from alcohol use. Each item is scored from zero to four with higher scores indicating greater risk. Items were summed, yielding a possible range of zero to 40. Cronbach’s alpha was.71.

*Alcohol-related problems* were evaluated using the Rutgers Alcohol Problem Index (RAPI; [55]). This 23-item scale examines the frequency with which participants have experienced negative consequences of drinking over the last three months (e.g. “Missed out on things because you spent too much money on alcohol”). Participants rated the frequency of each consequence on a 5-point scale from *never* (0) to *more than 10 times* (4). For the present study, two additional items were included to assess driving after drinking [56]. Cronbach’s alpha was.79.

#### Experimental manipulation

The “cross out e” task (also called the “stimulus detection task”) developed by Baumeister and colleagues [29] was used. Participants were provided with two typewritten pages of text and asked to cross off letters based on instructions that were either simple or complex. All participants started by crossing out the letter *e* whenever it appeared on the first page. For the second page, however, half of the participants were asked to complete the same task (i.e., cross off *e*’s) while the remaining half were asked to follow a complex set of instructions (i.e., cross out all *e*’s except when they were followed by a vowel in the same word or when a vowel is one letter away from the *e* in either direction). The complex set of instructions was intended to be more cognitively demanding than the original instructions.

#### Manipulation check

Following the “cross out e” task, participants completed a six-item manipulation check (adapted from [23,29,36,57,58]). Four items evaluated the extent to which participants found the task frustrating, unpleasant, difficult, and exhausting. The remaining two evaluated the amount of effort and self-control that participants felt the task required. Participants rated their agreement on a 25-point scale from *not at all* (1) to *very much* (25) [29,58]. One item (how exhausting participants found the task) was inadvertently not administered to the first 20 participants. Three additional items evaluated positive affect (α = 89) and three items evaluated negative affect (α =.86); items were rated on a 9-point scale from *not at all* (1) to *very much* (9); [59]). Urges to drink right now were also evaluated on a 9-point scale from *not at all* (1) to *very much* (9).

#### Taste test

Alcohol consumption was assessed using a modified taste-test procedure [60]. Participants were presented with three 350 ml beers (Rainier, Heineken, and Samuel Adams) and 350 ml of water in unlabeled cups. They were asked to rate each beer on a series of descriptors (e.g. taste, bitterness, strength) and to guess the brand of beer. Participants were told that the purpose of the taste test was to evaluate consumer preferences for beer. They were asked to take their time in tasting, to drink as much as they would like, and to rate each beer. They were told that they would have 10 minutes to complete the task. To provide an incentive to restrain drinking, participants were told by their experimenter that upon completion of the taste test, they would complete another RT task (i.e., an IAT), and that if their RTs were faster than their RTs on their previous IATs, they would win a prize. This approach was adapted from Ostafin and colleagues’ [23] procedures. After completing the task and unbeknownst to participants, experimenters measured and recorded the total volume (in ml) of beer consumed.

### Procedures

Study procedures were approved by the University of Washington’s institutional review board (Application #49370). The research team obtained contact information from the university’s registrar’s office for students in their third year or above who were at least 21 years of age (i.e., they provided a randomized list of 4971 individuals meeting these criteria). Prospective participants received an email invitation to participate in a lab-based study on alcohol taste preferences. If interested, they were asked to complete a phone or online screening to determine their eligibility (fluent in English, does not dislike beer). In addition, individuals needed to report at least one heavy drinking episode in the last 30 days (≥4/5 drinks in a single occasion for women/men). Seven hundred and fifty-three individuals completed screening; 428 individuals met screening criteria. Of those individuals, 256 completed a brief medical screening over the phone to establish that they did not have any health conditions (e.g., problematic drinking, allergies, conditions or medications) that would contraindicate participation in the alcohol taste test. Eligible individuals (*N* = 165) were then invited to come in for a lab session and asked not to drink alcohol or take drugs on the day of the session, not to drive to or from the laboratory, and to abstain from food or drink (other than water) for three hours before their session. The first 105 eligible individuals who attended the lab session were enrolled in the study.

Upon arrival at the lab session, participants were asked to provide a government ID (for proof of age and name) and lock up their belongings (to prevent distraction). They then completed written informed consent procedures. Female participants were then asked to complete a pregnancy test, as required by U.S. federal guidelines restricting pregnant women from participating in alcohol administration studies. Additionally, participants verified the accuracy of their medical screening. Finally, participants completed a blood alcohol reading using a hand-held breath alcohol tester (Alco-Sensor IV, Intoximeter, Inc.) to ensure that they began the study with a baseline blood alcohol concentration of 0.00 g/210 L. If experimenters found that participants had not complied with instructions (e.g., drove to session) or had a blood alcohol concentration above zero, they were rescheduled and sent home. This occurred one time (a participant reported taking over the counter cough medicine containing alcohol). If an inaccuracy in participants’ medical screening was found and indicated that participants could not consume alcohol safely or if a participant had a positive pregnancy test, they would not have been permitted to participate in the study (neither occurred for any study volunteer).

Participants then completed the computer-based baseline assessment, which included the IATs, explicit counterparts, implicit theory measure, OSPAN, alcohol consumption and problem measures, and other self-report measures not included in the present analyses (see S1 Table). All measures were presented in a randomized order with the exception of the OSPAN (presented first) and the IAT’s (evenly spaced throughout the assessment). This was intended to minimize participant fatigue. Four questions (e.g., “To answer this question correctly, you must answer ‘strongly agree’”) sure if necessary to cover additional demo questions not mentioned in paperional source) butmanipulation check. It is also incluto check participants’ attention and accuracy were interspersed throughout the measures. Participants were given feedback if they answered a check question incorrectly. Nine participants missed one question; two participants missed two questions; and no participants missed more than two questions. Upon completion, participants took a 10-minute break. At the end of the break, participants were randomly assigned to condition for the manipulation (more cognitively demanding—complex cross out *e* instructions—or less cognitively demanding—simple cross out *e* instructions). They then completed the manipulation and answered the manipulation check questions. Next, a second experimenter, who was blind to participants’ condition, completed the remaining procedures. That experimenter immediately presented participants with the 10-minute taste test. When they completed the taste test, or when 10 minutes were up, the experimenter removed the drinks and rating sheets. After 5 minutes had passed, experimenters took a blood alcohol reading, after which participants completed an additional drinking identity IAT. Participants were then informed that they had completed the study, were invited to retrieve their belongings, and were offered food, non-alcoholic beverages, and entertainment while they detoxed. Experimenters checked participants’ blood alcohol concentration again after 15 minutes had passed. If their blood alcohol reading was at or below 0.03 g/210 L, participants were debriefed and thanked for their participation. If the reading remained above 0.03 g/210 L, experimenters checked their blood alcohol every 10–20 minutes until that threshold was met. Upon leaving, participants were compensated $15 per hour spent in the lab, plus a $5 bonus for completing the second IAT (the latter is the prize that participants were told about during the taste test; it was not actually contingent on RT and all participants received the bonus). Mean length of participants’ stay in the lab was 129 minutes. If participants’ blood alcohol concentrations were above 0.0 g/210 L, they were also provided with a calculation of the amount of time that would pass before they reached 0.0 g/210 L and asked not to drive until that time.

### Data analysis

We used *t*-tests to compare the experimental conditions’ effects on baseline drinking, implicit and explicit measures of alcohol excite, alcohol approach, and drinking identity, working memory capacity, and implicit theories about willpower as well as on the manipulation check items. We used regression models to test whether experimental condition (coded as 0/1 with less cognitively demanding as 0) moderated the relation between implicit/explicit measures and drinking. We estimated three sets of regression models, one for each of the implicit/explicit measures (i.e., excite, approach, and identity). Within each set of models, one model evaluated whether condition moderated relationships between implicit alcohol associations/explicit measure counterparts and alcohol consumption, which we call the baseline model; a second model evaluated whether implicit theories about willpower further moderated the implicit/explicit x self-control interaction, which we call the implicit willpower model; and a third model evaluated whether working memory capacity (i.e., OSPAN score) further moderated the implicit/explicit x self-control interaction, which we call the working memory model. For all regression models, the outcome was total alcohol consumed. Because this measure was positively skewed, we used a natural log transformation. All continuous variables were entered as unstandardized variables and all models controlled for baseline alcohol consumption (DDQ score) and birth sex (dummy coded with 1 = women and 0 = men). For hypothesis tests, *α* was set at 0.05. We used a Bonferronni correction when evaluating significance for the manipulation checks (all reported *p*-values are uncorrected). All analyses were conducted using Stata version 15.

## Results

### Baseline data

Fifty-one participants were randomized to the more (vs. less) cognitively demanding condition (25 women; 50%) and 50 to the less cognitively demanding condition (22 women; 44%). Table 1 displays baseline means and SDs for the drinking measures, implicit and explicit measures for identity, approach, and excitement, working memory capacity, and willpower. They are presented as a function of condition; t-tests were conducted to test for potential baseline differences. None of the variables differed significantly at baseline. Table 2 contains zero-order correlations between study variables.

**Table 1:** Baseline data stratified by condition.

Condition				
Measure	More cognitively demanding *M* (*SD*)	Less cognitively demanding *M* (*SD*)	*t* value	*p* value
Drinks per week	13.25 (10.48)	14.20 (11.82)	0.43	0.67
Alcohol-related problems	4.18 (4.99)	5.58 (5.16)	1.39	0.17
Risk of AUD	7.43 (4.07)	8.90 (3.64)	1.91	0.06
Exp excite	15.84 (3.97)	15.00 (4.23)	-1.03	0.3
Exp approach	5.66 (1.54)	5.92 (1.21)	0.96	0.34
Exp identity	1.98 (0.96)	1.92 (0.95)	-0.34	0.74
Imp excite^a^	0.04 (0.51)	0.05 (0.38)	0.15	0.88
Imp approach^b^	-0.06 (0.38)	-0.04 (0.32)	0.36	0.72
Imp identity^c^	0.19 (0.39)	0.10 (0.39)	-1.14	0.26
Working memory^d^	50.10 (11.75)	47.86 (12.10)	-0.90	0.37
Imp willpower	2.47 (0.74)	2.76 (0.90)	1.72	0.09

*N* = 101;

^a^ *N* = 100;

^b^ *N* = 97;

^c^ *N* = 98;

^d^ *N* = 91.

Drinks per week = number of U.S. standard drinks consumed in a typical week, as reported on the Daily Drinking Questionnaire. Alcohol-related problems = scores on the Rutgers Alcohol Problem Index; higher scores = more problems. Risk of AUD = score on the Alcohol Use Disorders Identification Test; higher scores = greater risk of an alcohol use disorder. Exp excite = mean score on the enhancement subscale of the Drinking Motives Questionnaire; higher scores = stronger inclination to drink alcohol. Exp approach = mean score on the inclined/indulgent subscale of the Approach and Avoidance of Alcohol Questionnaire; higher scores = stronger inclination to drink alcohol. Exp identity = dichotomized mean score on the Alcohol Self-Concept Scale; 0 = absolutely no endorsement of drinking identity, 1 = anything other than no endorsement. Imp excite/approach/identity: scores on the alcohol excite, alcohol approach, or drinking identity IATs, respectively; higher scores = stronger association between the constructs indicated (e.g., alcohol and excitement, drinking and the self). Working memory = score on the Operation Span Task; higher scores = greater working memory capacity. Imp willpower = mean score on Implicit Theories about Willpower; higher scores = stronger belief that willpower is an unlimited (vs. limited) resource.

**Table 2:** Correlation matrix.

Measure	1	2	3	4	5	6	7	8	9	10	11	12
1. Alcohol-related problems	1											
2. Risk of AUD	0.64***	1										
3. Drinks per week	0.55***	0.65***	1									
4. Imp identity	0.11	0.18	0.31**	1								
5. Imp excite	-0.03	0.02	0.08	0.11	1							
6. Imp approach	0.06	0.18	0.09	0.13	0.33**	1						
7. Exp identity	0.33**	0.34**	0.26**	0.26**	0.16	0.10	1					
8. Exp excite	0.27**	0.30**	0.37***	0.26**	0.12	0.03	0.40***	1				
9. Exp approach	0.32**	0.44***	0.42***	0.25*	0.04	0.20	0.22*	0.24*	1			
10. Imp Willpower	-0.01	0.01	0.16	0.01	0.06	-0.01	-0.09	-0.05	0.09	1		
11. Working memory	0.15	-0.02	0.09	-0.09	-0.03	-0.12	-0.07	0.01	0.02	0.10	1	
12. Taste test	0.37***	0.29**	0.33**	0.08	0.01	-0.01	0.17*	0.24*	0.17	-0.09	-0.08	1

*N* = 101. Samples sizes for each correlation range from 87–101 due to missing data and/or invalid scores on IATs or the OSPAN. Alcohol-related problems = scores on the Rutgers Alcohol Problem Index; higher scores = more problems. Risk of AUD = score on the Alcohol Use Disorders Identification Test; higher scores = greater risk of an alcohol use disorder. Drinks per week = number of U.S. standard drinks consumed in a typical week, as reported on the Daily Drinking Questionnaire. Imp identity/excite/approach: scores on the drinking identity, alcohol excite, or alcohol approach IATs, respectively; higher scores = stronger association between the constructs indicated (e.g., alcohol and excitement, drinking and the self). Exp identity = dichotomized mean score on the Alcohol Self-Concept Scale; 0 = absolutely no endorsement of drinking identity, 1 = anything other than no endorsement. Exp excite = mean score on the enhancement subscale of the Drinking Motives Questionnaire; higher scores = stronger inclination to drink alcohol. Exp approach = mean score on the inclined/indulgent subscale of the Approach and Avoidance of Alcohol Questionnaire; higher scores = stronger inclination to drink alcohol. Imp willpower = mean score on Implicit Theories about Willpower; higher scores = stronger belief that willpower is an unlimited (vs. limited) resource. Working memory = score on the Operation Span Task; higher scores = greater working memory capacity. Taste test = total ml of beer consumed during taste test.

* *p* < 0.05,

** *p* < 0.01,

*** *p* < 0.001

### Manipulation check

Table 3 displays means, standard deviations, and *t*-tests for the manipulation check variables. Contrary to expectations that more (vs. less) complex instructions on the cross out *e* task would require more self-control, more effort, be more difficult, and be more exhausting, there were no significant differences in participants’ ratings of the task. Thus, there was little evidence that the more (vs. less) cognitively demanding condition was evaluated as such by study participants. Items assessing affect related to the task (e.g., frustration, pleasantness) and participants’ mood (positive affect, negative affect) were not expected to differ as a function of condition as the task was not intended to affect mood. The only significant difference between conditions was for "Frustrating,” with greater frustration reported by participants who completed the more (vs. less) complex cross out *e* task (*t* = 2.05, *p* = 0.04). This difference was not significant following a Bonferroni correction for multiple tests.

**Table 3:** Manipulation check.

Condition				
Measure	More Cognitively Demanding *M* (*SD*)	Less Cognitively Demanding *M* (*SD*)	*t* value	*p* value
Frustration	13.33 (7.36)	10.56 (6.11)	2.05	0.04
Effort^a^	13.06 (5.88)	12.49 (6.39)	0.46	0.64
Unpleasantness	14.41 (8.12)	13.48 (7.00)	0.62	0.54
Difficulty	7.94 (6.11)	7.74 (5.66)	0.17	0.86
Self-control required	12.06 (6.77)	11.54 (6.75)	0.39	0.70
Exhaustion^b^	11.73 (7.17)	10.07 (6.55)	1.09	0.28
Negative affect	2.65 (1.45)	2.60 (1.63)	0.17	0.86
Positive affect	4.57 (1.28)	4.97 (1.67)	-1.35	0.18
Urge	2.55 (2.13)	2.70 (2.17)	-0.35	0.72

*N* = 101;

^a^ *N* = 100;

^b^ *N* = 82 (this item was accidently omitted for the first 20 participants).

### Alcohol consumption as a function of condition and implicit and explicit drinking identity, alcohol excite, and alcohol approach

Taste test alcohol consumption averaged 258 ml (*SD* = 191 ml, range 24 to 1039 ml). Tables 4, 5 and 6 provide the regression results for the models (referred to as baseline models in the tables) evaluating the association between each IAT and its explicit measure counterpart and alcohol consumption as a function of experimental condition. The results were not consistent with the hypothesis that condition would moderate the relationship between each IAT/explicit measure and alcohol consumption. Specifically, the two-way interaction between IAT scores and condition was not significant for any of the IATs, and the two-way interaction between explicit measure scores and condition was also not significant for any of the explicit measures (all *p* >.05).

**Table 4:** Drinking identity models.

	Baseline model		Imp willpower		Working memory	
	*B*	*SE*	*B*	*SE*	*B*	*SE*
Drinks per week	0.01	0.01	0.01	0.01	0.02	0.01
Condition	0.09	0.29	1.15	1.04	-4.78	2.48
Imp identity	-0.02	0.29	-0.03	0.95	0.45	2.12
Exp identity	0.30	0.24	0.29	0.74	-3.46	2.09
Condition x Imp identity	-0.13	0.39	-0.49	1.48	-1.92	3.63
Condition x Exp identity	-0.16	0.34	-1.08	1.2	5.02	2.57
Sex	0.52***	0.15	-0.53**	0.16	-0.42**	0.17
Imp willpower			-0.14	0.19		
Condition x Imp willpower			-0.42	0.37		
Imp identity x Imp willpower			0.002	0.32		
Exp identity x Imp willpower			-0.01	0.24		
Condition x Imp identity x Imp willpower			0.16	0.52		
Condition x Exp identity x Imp willpower			0.35	0.43		
Working memory					-0.08*	0.03
Condition x Working memory					0.10	0.05
Imp identity x Working memory					-0.01	0.04
Exp identity x Working memory					0.07	0.04
Condition x Imp identity x Working memory					0.03	0.07
Condition x Exp identity x Working memory					-0.10	0.05
Intercept	5.20***	0.24	5.60***	0.62	9.30***	1.88
*N*	98		98		88	

*B* = unstandardized regression coefficient. Drinks per week = number of U.S. standard drinks consumed in a typical week, as reported on the Daily Drinking Questionnaire. Condition = experimental condition and was coded 0 = less cognitively demanding, 1 = more cognitively demanding. Imp identity = score on the drinking identity IAT; higher scores = stronger association between drinking and the self. Exp identity = dichotomized mean score on the Alcohol Self-Concept Scale; 0 = absolutely no endorsement of drinking identity, 1 = anything other than no endorsement. Sex = birth sex and was coded 0 = male, 1 = female. Imp willpower = mean score on Implicit Theories about Willpower; higher scores = stronger belief that willpower is an unlimited (vs. limited) resource. Working memory = score on the Operation Span Task; higher scores = greater working memory capacity. The outcome is the log of taste test alcohol consumption.

* *p* < 0.05,

** *p* < 0.01,

*** *p* < 0.001

**Table 5:** Alcohol excite models.

	Baseline model		Imp willpower		Working memory	
	*B*	*SE*	*B*	*SE*	*B*	*SE*
Drinks per week	0.01	0.01	0.02*	0.01	0.02*	0.01
Condition	-0.21	0.58	2.49	2.13	-6.35*	2.83
Imp excite	0.07	0.29	0.73	0.86	-1.31	1.33
Exp excite	0.01	0.03	-0.02	0.08	-0.17	0.14
Condition x Imp excite	-0.1	0.35	-1.91	1.06	-0.96	2.12
Condition x Exp excite	0.01	0.04	-0.15	0.14	0.36	0.18
Sex	-0.43**	0.16	-0.41*	0.16	-0.36*	0.17
Imp willpower			-0.21	0.4		
Condition x Imp willpower			-1.25	0.81		
Imp excite x Imp willpower			-0.22	0.29		
Exp excite x Imp willpower			0.01	0.03		
Condition x Imp excite x Imp willpower			0.72	0.39		
Condition x Exp excite x Imp willpower			0.07	0.05		
Working memory					-0.08	0.04
Condition x Working memory					0.12*	0.06
Imp excite x Working memory					0.02	0.03
Exp excite x Working memory					0.01	0.01
Condition x Imp excite x Working memory					0.02	0.04
Condition x Exp excite x Working memory					-0.01	0.01
Intercept	5.19***	0.42	5.85***	1.26	8.83***	2.24
*N*	100		100		90	

*B* = unstandardized regression coefficient. Drinks per week = number of U.S. standard drinks consumed in a typical week, as reported on the Daily Drinking Questionnaire. Condition = experimental condition and was coded 0 = less cognitively demanding, 1 = more cognitively demanding. Imp excite: score on the alcohol excite IAT; higher scores = stronger association between alcohol and excitement. Exp excite = mean score on the enhancement subscale of the Drinking Motives Questionnaire; higher scores = stronger inclination to drink alcohol. Sex = birth sex and was coded 0 = male, 1 = female. Imp willpower = mean score on Implicit Theories about Willpower; higher scores = stronger belief that willpower is an unlimited (vs. limited) resource. Working memory = score on the Operation Span Task; higher scores = greater working memory capacity. The outcome is the log of taste test alcohol consumption.

* *p* < 0.05,

** *p* < 0.01,

*** *p* < 0.001

**Table 6:** Alcohol approach models.

	Baseline model		Imp willpower		Working memory	
	*B*	*SE*	*B*	*SE*	*B*	*SE*
Drinks per week	0.01	0.01	0.02	0.01	0.02	0.01
Condition	0.54	0.67	0.42	2.62	-2.33	3.20
Imp approach	0.09	0.33	1.08	1.28	0.05	2.00
Exp approach	0.11	0.09	0.16	0.36	-0.08	0.40
Condition x Imp approach	-0.39	0.44	-1.15	1.65	0.26	2.47
Condition x Exp approach	-0.1	0.11	-0.03	0.44	0.34	0.56
Sex	-0.4*	0.16	-0.48**	0.17	-0.34	0.18
Imp willpower			-0.03	0.76		
Condition x Imp willpower			0.03	0.96		
Imp approach x Imp willpower			-0.38	0.46		
Exp approach x Imp willpower			-0.02	0.12		
Condition x Imp approach x Imp willpower			0.29	0.62		
Condition x Exp approach x Imp willpower			-0.03	0.16		
Working memory					-0.34	0.18
Condition x Working memory					0.06	0.06
Imp approach x Working memory					-0.01	0.04
Exp approach x Working memory					0.01	0.01
Condition x Imp approach x Working memory					-0.01	0.05
Condition x Exp approach x Working memory					-0.01	0.01
Intercept	4.68***	0.56	4.82*	2.24	6.30**	2.28
*N*	97		97		87	

*B* = unstandardized regression coefficient. Drinks per week = number of U.S. standard drinks consumed in a typical week, as reported on the Daily Drinking Questionnaire. Condition = experimental condition and was coded 0 = less cognitively demanding, 1 = more cognitively demanding. Imp approach: score on the alcohol approach IAT; higher scores = stronger association between alcohol and approach. Exp approach = mean score on the inclined/indulgent subscale of the Approach and Avoidance of Alcohol Questionnaire; higher scores = stronger inclination to drink alcohol. Sex = birth sex and was coded 0 = male, 1 = female. Imp willpower = mean score on Implicit Theories about Willpower; higher scores = stronger belief that willpower is an unlimited (vs. limited) resource. Working memory = score on the Operation Span Task; higher scores = greater working memory capacity. The outcome is the log of taste test alcohol consumption.

* *p* < 0.05,

** *p* < 0.01,

*** *p* < 0.001

Although not reported in Tables 4–6, we also ran the models without the two-way interactions to test whether there was a condition effect on alcohol consumption or whether there was a significant relation between IAT/explicit measure and alcohol consumption. None of those relationships were significant.

In all of the models tested, the only variable that significantly predicted alcohol consumption in the taste test was sex, with men consuming more alcohol than women.

### Models evaluating implicit theories about willpower and working memory capacity as additional moderators

Finally, we ran models that tested whether implicit theories about willpower and working memory capacity might moderate the relationship between identity, excite, and approach, and condition (see Tables 4–6). The results for the three-way interactions were similar to the two-way interactions described above. Specifically, neither the measure of implicit theories about willpower nor the measure of working memory capacity was a significant moderator of the two-way interactions between condition and IAT scores or between condition and the explicit measure counterparts. We note one instance of main effects of working memory capacity (in the model containing drinking identity variables) and condition (in the model containing alcohol excite variables) and one instance of a condition by working memory capacity interaction (in the model containing alcohol excite variables). These effects were not relevant to central aims of the study (and should we have imposed corrections for multiple tests, they would not have survived them), thus, we did not interpret those effects.

## Discussion

We tested whether engaging in a more (vs. less) cognitively demanding task and having an incentive to exert self-control over drinking moderated the relationship between implicit processes (specifically, implicit measures of alcohol-related associations) and alcohol consumption in the laboratory. Our goals in doing so were to test theory-driven hypotheses about factors that are posited to moderate the influence of implicit processes on alcohol consumption, to test whether they hold for single drinking occasions (vs. the aggregated self-reported drinking behavior that is more commonly studied), and to test these hypotheses across three implicit measures of alcohol-related associations that have been shown to predict problematic drinking in U.S. college students. Given the larger ego-depletion controversy, we also evaluated two additional moderators of the hypothesized relationship—measures of implicit theories about willpower and working memory capacity. Contrary to predictions, none of the proposed relationships was observed in this study.

With respect to implicit measures of alcohol-related associations, findings were not only null with respect to whether the combination of a (more vs. less) cognitively demanding task and incentive for self-control moderated the relation between those associations and taste test consumption, they were also null with respect to a relation between those associations and taste test consumption. In other words, there was no evidence that IAT scores were associated with taste test consumption. Why might that have occurred? There are a number of possible factors, including the properties of IAT in general and of the particular IATs tested. Regarding the former, implicit measures are generally lower in internal consistency than explicit measures, which can limit their predictive validity. At the same time, the IAT itself has among the best psychometrics when compared to other implicit measures [61] and has greater predictive validity when used in a standard format [62], as was done in the current study. Moreover, the format, stimuli, and scoring for the current study’s IATs were identical to the IATs that we have used in previous studies with U.S. college students [19,20,27]. Further, the internal consistencies for study IATs were similar to our previous studies, including those conducted in the lab [19,21,27] and online [20], with the possible exception of the alcohol approach IAT (internal consistency in the current study was.43 as compared to.48.-.58 in our other studies). Mean IAT scores for the sample were also generally consistent with our previous studies, particularly those that recruited heavier drinkers [27]. Finally, while lower in magnitude than some studies (potentially due to restricted range due to study inclusion and exclusion criteria), results provided some evidence of positive associations between IAT scores (particularly for the drinking identity IAT) and self-reported drinking outcomes on the questionnaires. Thus, there was no obvious indication in the current study that the IATs’ stimuli and format, their internal consistencies, the average observed IAT scores, or the IATs’ relations with self-reported drinking were atypical for a U.S. college student sample of heavy social drinkers.

What other factors might account for our null findings of a relation between the IAT scores and consumption during the taste test? Close review of prior studies that evaluated the effects of self-control manipulations on laboratory drinking and that used IATs [23,25] revealed that those studies matched the alcoholic beverage stimuli in the IATs (beer) with the alcoholic beverages consumed in the taste test (beer). Given findings [62,63] that greater correspondence between the content of IATs and criterion (whether explicit measures or behavior) result in larger IAT-criterion correlations, it is possible that our null findings stemmed at least in part from an imperfect match between our IAT stimuli (i.e., images of a variety of alcoholic beverages that participants selected for the alcohol approach and alcohol excite IATs, or words describing drinkers and drinking for the drinking identity IAT) and the alcoholic beverages we provided in the taste test (beer only). Future studies of laboratory drinking might consider matching IAT stimuli to alcohol beverages provided and/or testing the consequences of having matching or more general IAT stimuli. It seems unlikely to us, however, that the associations and beverages need to be so identical and this would suggest the prediction is not robust to minor deviations. Additionally, when designing the study, we based our power calculations on research that suggested a small to medium effect size for the interactions (e.g., *r* between 0.2 to 0.3 or about a 7% increase in *r*^2^). More recent research suggests that effects from the ego-depletion literature in particular are likely overestimated in older research. Consequently, our study may have been underpowered.

Two other methodological factors seem more likely to account for the lack of relation between alcohol-related associations and drinking: a weak experimental manipulation and ecological validity issues with the alcohol taste test. Evidence from previous studies of positive associations between alcohol-related IAT scores or other implicit measures and laboratory alcohol consumption occurred in the context of experimental manipulations that, based on analysis of manipulation checks, were successful [23–25,27,28]. In contrast, when the manipulation check data were analyzed in the current study, there was no evidence that this manipulation was successful. Despite using a validated measure [29,36], the manipulation checks revealed essentially no differences in indices related to effort, difficulty, or self-control. This is particularly unfortunate because it leaves open the question of whether circumstances that are more (vs. less) cognitively demanding and include incentives to control one’s drinking moderates the relation between alcohol-related associations and drinking. Published studies [23–25] do not provide a complete answer to this question because they also included mood inductions and mood suppression. The picture is further complicated by studies that have evaluated mood (only) as a moderator of implicit alcohol-related measures and alcohol consumption in the laboratory *and* have found some evidence for the hypothesized relation, particularly in the context of implicit measures related to alcohol and affect [27,28]. Finally, we note the possibility that the OSPAN itself—administered at baseline to all participants—could have essentially functioned as a task that required substantial cognitive efforts and heavily taxed participants’ self-control. The OSPAN is indeed a measure that involves substantial cognitive effort (at least two of the authors on this paper have had difficulty achieving a valid score!). We attempted to minimize potential effects of completing the OSPAN as well as any and all of the baseline measures by providing participants with a 10-minute break where they had the opportunity to use the restroom, take a short walk, and/or draw or complete coloring pages. Collectively, these factors make clear the need for future research that uses a more powerful manipulation and does so under conditions where control is not already taxed.

With respect to ecological validity of the taste test, we note the long-standing concern about the ecological validity of laboratory-based alcohol administration procedures [64], particularly the mismatch between how college students typically drink (i.e., with other people) versus how they are asked to drink in the lab (i.e., alone). Thus, a critical issue is whether the relation between alcohol-related associations and single drinking sessions would be stronger with improvements to ecological validity (i.e., laboratory alcohol administration procedures with dyads or groups or studies using ecological momentary assessment). At the same time, we note key null findings from other studies [65] that included dyads (with a participant and confederate) and groups (with a participant and his/her friends) that found no evidence of a positive relation between alcohol-related associations and drinking. We also considered whether the range and variance in taste test alcohol consumption was limited. However, both the average and range of taste consumption are not dissimilar to those in other studies [23,27] so there do not appear to be issues with respect to restricted range. A final possibility could be that our instructions for the taste test failed to elicit a strong motive to exert self-control and restrain drinking. Though we told participants upon completion of the taste test that they would complete another RT task and could win a prize if their RTs were faster than on their previous RT tasks, we did not make the link between drinking alcohol and slowed RT explicit (whereas Ostafin et al.’s [23] instructions did).

Finally, we note the null findings for key additional moderators, namely implicit theories about willpower and working memory capacity. Though selected to minimize concerns related to the ego-depletion controversy, they, too, failed to demonstrate predicted relations. It may be that we were simply underpowered for the posited three-way interactions. We also note that, in the context of the failed manipulation, one would still expect two-way interactions between IAT scores and working memory capacity (and two-way interactions with the explicit measure counterparts and working memory capacity). These were not observed. OSPAN scores ranged from 0 to 60, but the distribution was negatively skewed and approximately 50% of participants received scores of 55 or 60, which could have an impact on findings.

Beyond these possible methodological and statistical explanations, we also must consider the possibility that the null findings accurately reflect that implicit measures of alcohol-related associations and their explicit measure counterparts predict long-term or aggregated drinking patterns (as evident in prior studies) but not in-the-moment drinking instances. It may be that current measures of these associations and/or the underlying associations themselves are limited in their ability to predict drinking in-the-moment (vs. aggregated drinking sessions over time). Additionally, it is often presumed that implicit measures index more trait-like representations [17,66,67], but theories propose and empirical findings indicate that such measures can also index state-dependent effects [68–70]. Thus, it is also possible that having participants complete the IATs closer in time to the alcohol taste test would produce stronger implicit-behavior correlations because the IAT scores (and the presumed underlying alcohol associations) are at least somewhat state-dependent.

### Strengths, limitations, and future directions

Despite null findings, the study has important strengths. It is one of the few experimental studies that evaluates moderators of implicit processes and includes actual alcohol administration. Our findings, even amidst a failed manipulation, indicate that there are important, unresolved questions about when and how implicit measures are related to discrete drinking sessions. Second, it includes manipulation checks that assessed the manipulation, which is crucial because one (of the many) concerns in the ego depletion controversy is the number of studies that do not include or report manipulation check items [31]. In the context of this controversy and the larger movement toward more transparent, open science practices, it is essential to evaluate the effects of a manipulation, to report those effects, and more generally, to publish null findings. Third, we used implicit measures, their explicit counterparts, a manipulation, and a taste test procedure that have been previously validated, which adds to the strength of the study.

Our study was also not without limitations. Most importantly, the manipulation was weak: the two conditions did not appear to differ with respect to being more (vs. less) cognitively demanding. Future studies will need to implement more powerful manipulations. Second, the lack of correspondence between the IAT stimuli and taste test raises questions about whether a closer match would have yielded different results with respect to their relation. Relatedly, while the internal consistencies reported for the study’s IATs were consistent with published studies [19,20,27], they are low, which limits their ability to predict behavior. Third, while there are many strengths to laboratory alcohol administration procedures, including alcohol taste tests, they also lack ecological validity. Fourth, generalizability of findings to other populations is unclear.

## Conclusion

We evaluated the relation between validated implicit measures of alcohol-related associations and drinking in the laboratory in a lab-based study with a manipulation that was intended to be more (vs. less) cognitively demanding and provide an incentive to control one’s drinking. Secondary aims included evaluating individual differences related to self-control (implicit theories about willpower and working memory capacity) that might further moderate these relationships. Despite the use of validated measures, the study yielded null findings, including null findings for the relationship between alcohol-related associations and drinking in the laboratory. Findings indicate the importance of future research that can clarify whether and/or how implicit measures are related to discrete drinking sessions.

## Supporting information

S1 Table Additional measures administered during computer-based baseline assessment. (DOCX)
