# Supplementary material for: Self-control, implicit alcohol associations, and the (lack of) prediction of consumption in an alcohol taste test with college student heavy episodic drinkers
Source: PLoS One. 2019 Jan 9;14(1):e0209940. doi: 10.1371/journal.pone.0209940 (PMC6326486; doi:10.1371/journal.pone.0209940)
Supplement: S1 Table — (DOCX) [file pone.0209940.s001.docx]

**S1 Table. Additional Measures Administered During Computer-based Baseline Assessment**

| **Measure** | **Author(s)** |
| --- | --- |
| After experiment plans | - Created for this study |
| Alcohol Craving Questionnaire - Short Form - Revised (ACQ-SF-R) | - Singleton, Tiffany, & Henningfield (1995) |
| Alcohol Interpretation Bias | - Chow, Portnow, Zhang, Salemink, Wiers, & Teachman (2018) |
| Alcohol Purchase Task (APT) | - Murphy & MacKillop (2006) |
| Alcohol-related compensatory behaviors | - Hunt & Forbush (2016) |
| Brief Big 5 | - Rammstedt & John (2007) |
| Drinking Identity Centrality | - Lindgren, Ramirez, Namaky, Olin, & Teachman (2016) |
| Implicit Theory of Personality | - Levy, Stroessner, & Dweck (1998) - Plaks, Stroessner, Dweck, & Sherman (2001) - Chiu, Hong, & Dweck (1997) |
| Implicit Theory of Personality - Alcohol Mindset and Drinking Problem Mindset Subscales | - Created for this study - [Adapted from Implicit Theory of Personality sources above] |
| Quantity/Frequency Scale (QF) | - Baer (1993) - Marlatt, Baer, & Larimer (1995) |
| Ruminative Response Scale (RRS-10) | - Treynor, Gonzalez, & Nolen-Hoeksema (2003) |
| Self-Control Scale (SCS) | - Tangney, Baumeister, & Boone (2004) |
| Semantic differential items | - [Adapted from] Nosek, Greenwald, & Banaji (2007) |
| State Motivation for Alcohol Control (SM-A) | - Robinson, Kavanagh, Connor, May, & Andrade (2016) |
| Substance Use Risk Profile Scale (SURPS) | - Woicik, Stewart, Pihl, & Conrod (2009) |
